# Hearing own or other’s name has different effects on monotonous task performance

**DOI:** 10.1371/journal.pone.0203966

**Published:** 2018-09-26

**Authors:** Kosuke Kaida, Sunao Iwaki

**Affiliations:** Automotive Human Factors Research Center, National Institute of Advanced Industrial Science and Technology (AIST), Tsukuba, Ibaraki, Japan; University of Wuerzburg, GERMANY

## Abstract

We examined the effect of hearing two types of self-relevant words, one’s own name and the name of others, on vigilant attention, arousal, and subjective sleepiness during performing the Psychomotor Vigilance Test (PVT). Participants performed the PVT under three experimental conditions, (a) hearing own full name (high self-relevant condition), (b) hearing other’s name (low self- relevant condition) and (c) the control condition with no stimuli. Participants heard the names every 20 sec. Self-relevance was assessed before the experiment using the self-relevance scale. The results of the behavioral effects are relatively small and not consistently supported by all of the performance indicators. A tentative conclusion, based on the overall pattern of results, is that (1) arousal increased by hearing a name, regardless of its self-relevance, and (2) hearing less self-relevant stimuli such as other’s name had a distractive effect on ongoing task performance, although it increased arousal, being aware that further experiments are urgently necessary.

## Introduction

Hearing sounds during a monotonous task is known to facilitate performance even if the stimuli are neither relevant nor informative for executing the task [1]. A classical study by Fox and Embrey (1972) reported that background music facilitated productivity during repetitive work [2]. Poulton (1979) reported that this facilitation effect may be related to arousal increase [3]. On the other hand, other studies which examined the effect of sound presented during the inter-stimulus interval (i.e., during the moment of preparatory processes to respond) on task performance suggest that the sounds disturb performance, because attentional resources are consumed by the task-irrelevant sound [4, 5]. It has been also reported that task-irrelevant sounds disturb performance [6].

What is then the essential factor of sounds that influences performance? In the present study, we focused on self-relevance of sounds and examined the effects of hearing either high (own name) or low (other’s name) self-relevant sounds on performance.

A typical example of a self-relevant stimulus is a person’s name. When we hear our own name, we automatically focus our attention on the sounds, and arousal is immediately increased to prepare for a self-relevant event that might occur with a high probability following the stimulus. It is also known that such stimuli induce a nonspecific state of readiness to respond [7], which facilitates the processing of any upcoming stimulus [8]. Moreover, a previous study reported that a person’s name compared to the no-sound, control condition, increased arousal level measured by the alpha power of EEG (7–14 Hz) and improved performance in the Psychomotor Vigilance Test (PVT), while no significant effect was observed on subjective sleepiness [9]. Alpha power density is known to be negatively associated with reaction times in PVT, that is, when the observed alpha power is small, the measured reaction time is short [10].

Therefore, calling a person’s name could be a practical strategy for increasing the arousal level and improving performance during monotonous tasks such as a night watch, or driving. In most actual work situations, we usually hear not only our own names but also names of other people which are much less self-relevant, such as names of celebrities mentioned on the radio when driving. Hearing names could facilitate task performance, whereas attentional orientation induced by less self-relevant sounds might be a distractive factor that deteriorates performance. The distractive effects of sounds have been previously reported [6], which might be due to lower self-relevance of stimuli compared to own names. Regardless of potentially critical effects of self-relevance of stimuli on the performance of monotonous tasks, only a few studies have explored this issue.

We examined the effect of less self-relevant stimuli on physiological arousal and vigilance by using two types of stimuli, the participant’s name as the self-relevant stimulus and the name of an acquaintance as the less self-relevant stimulus. The study was designed to investigate the facilitative or distractive effect of less self-relevant stimuli compared to more self-relevant stimuli on performance and physiological arousal.

## Methods

### Participants and design

Participants were 30 healthy, native Japanese speakers aged 20–34 years (mean = 23.3, standard deviation (SD) = 3.61, 11 women). All participants met the following criteria: (1) had a normal sleep-wake cycle, classified as intermediate type by the Morningness–Eveningness (ME) questionnaire [11, 12], (2) had no experience of shift work within the 3 months prior to the experiment, (3) had not traveled to a different time zone within the 3 months prior to the experiment, (4) had not used any medication, (5) had not used tobacco products, and (6) had a body mass index less than 25 (calculated as weight in kilograms divided by the square of the height in meters; BMI). Participants’ scores were as follows: ME, 49.2 (SD = 7.39); and BMI, 21.0 (SD = 2.58) kg/m^2^. They reported they had slept for 470.6 (SD = 71.24) min on the night before the experiment day. Participants were paid for taking part in the study.

**Fig 1:**
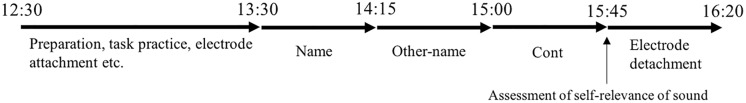
Time schedule of the experiment. Name = name condition, Other-name = other’s name condition, and Cont = control condition (no sound). The order of the conditions was counterbalanced among the participants.

Participants arrived at the laboratory at 12:30 and received a full explanation of the procedure. Then, they signed an informed consent document. We confirmed that all the participants had eaten lunch before arriving at the laboratory. The experiment began at 13:30 after attaching electroencephalogram (EEG) and electrooculogram (EOG) electrodes and conducting a practice session of performing the task. The time schedule of the experiment is shown in Fig 1.

**Fig 2:**
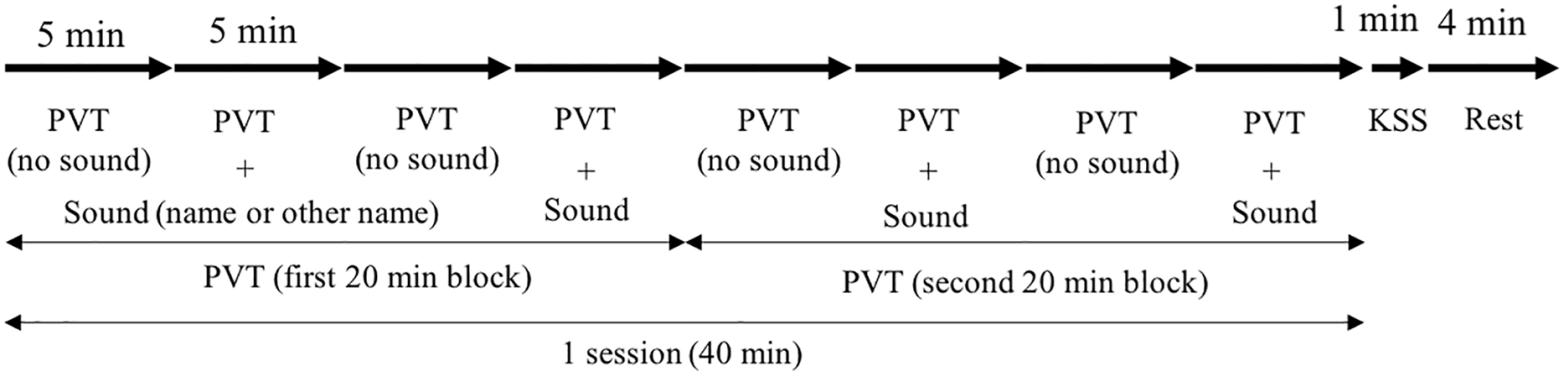
Sample of test session. KSS = Karolinska Sleepiness Scale, PVT = Psychomotor Vigilance Test. Visual stimuli for PVT were presented with random inter-stimulus intervals between 2–10 sec, while a sound was presented every 20 sec in the stimuli epochs. The order of the conditions was counterbalanced among the participants.

In one test session, participants conducted the Psychomotor Vigilance Test (PVT) [13] for 40 min without a break (see Fig 2). During the first 5 min of the PVT session, which was the no stimuli epoch, participants conducted the task in silence. In the next 5 min, which was the stimuli epoch, participants were exposed to an auditory stimulus every 20 sec while performing PVT in the name and other’s name conditions, or they were in the silent condition while performing PVT. The same procedure was repeated four times (i.e., eight 5 min epochs) in one experimental condition. They took a short break after each 40 min session, only when necessary. Participants conducted three test sessions and completed all the experimental conditions, which took them approximately 120 min.

Participants took part in the three following experimental conditions with the order of conditions counterbalanced among participants: (1) the own name condition (Name), (2) other’s name condition (Other-name), and (3) no-stimuli control condition (Cont). The order of 40-min sessions of the three conditions were counterbalanced, in which 6 different patterns (3*2 patterns) were allocated to 30 participants. In the Name condition, the participant’s own full name was played every 20 sec during the stimuli epochs. In the Other-name condition, participants heard other person’s name played every 20 sec during the stimuli epochs. The name played in the Other-name condition was decided for each participant and controlled to have less self-relevance by the following procedure. First, the participants scored the self-relevance of the 20 personal names provided by the experimenter using a 9-point Likert scale to identify the level of self-relevance reference of listed names. Participants were then asked to give a personal name that was not on the list, which was known to them and had a self-relevance score equal to the listed names with a score of 5 out of 9 in the scale (own names were scored around 8–9 in the scale). The name given by each participant in this procedure was used as the less self-relevant stimulus in the Other-name condition. In the Cont condition, no stimuli were presented during the 40-min task.

In the Name and Other-name conditions, participants performed PVT without any auditory sound for 5 min in the “PVT (no sound)” epoch (see Fig 2), followed by the “PVT + sound” epoch, in which participants continued PVT with hearing the sounds (name or other’s name) for 5 min, and the identical procedure was conducted 4 times (40 min for one session). We only used the data of the four “PVT + sound” epochs for analysis. The first two epochs were combined into the first block and another two epochs into the second block to increase data stability. No auditory stimuli were presented in the Cont condition and participants continued PVT in silence for 40 min. The purpose of inserting no sound epochs in both the Name and Other-name conditions was to avoid the habituation effect of sounds. The stimuli in both the Name and Other-name conditions were developed using artificial text reading software with a female voice. Each stimulus lasted approximately 2 sec (depending on the length of the name played). Sound pressure level was set at 70 dB measured at the ear level. The inter-stimulus interval between the PVT visual stimulus and the sound was not controlled (i.e., PVT stimuli were presented at random ranged between 2–10 sec while sounds were presented exactly every 20 sec).

Ethical considerations regarding the experimental protocol were reviewed and approved by the ethical review board at the National Institute of Advanced Industrial Science and Technology (AIST) of Japan, according to the principles stated in the Declaration of Helsinki.

### Psychomotor Vigilance Test (PVT)

The PVT uses a visual reaction time paradigm with inter-stimulus intervals ranging from 2 to 10 sec [13]. Participants were instructed to monitor a red square shown in the device display (PVT-192, Ambulatory Monitoring, Inc., Ardsley, NY) and press a response button on the device as soon as a red number counting down by milliseconds appeared within the square. The counter stopped when the participants responded, and the reaction time in milliseconds was displayed for one sec as feedback to the participant. Responses within 100 msec received warning signals (“FS”; false start) for one sec. The False Starts were treated as timeout trials, which continued on to the next trial.

The data of the first 30 sec in each epoch were omitted. Data with a standard deviation of 2.5 or higher (SD of all the RT data in the three conditions) than the mean was omitted when calculating the mean reaction times (RT). The number of data deviating from SD by 2.5 or more was counted as lapses. The reaction time coefficient of variation (i.e., CV) was calculated by dividing the individual standard deviation of an individual by the mean of that individual. By this means, a percentage-based measure is obtained that can directly be interpreted as to index participant’s performance fluctuations [14].

### Electroencephalogram (EEG)

Electrodes were attached at the Cz scalp site for EEG referenced to linked electrodes at the earlobes, and outside both canthi for EOG. The sampling rate was 1000 Hz (24-bit AD conversion), and the time constants were 0.3 sec for the EEG and 3.2 sec for the EOG. Electrode impedance was maintained below 5 kΩ. The low-pass filter was set at 30 Hz. Electrophysiological data were recorded with a portable digital recorder (PolymateV AP5148, Digitex Laboratory Co., Ltd., Japan).

Alpha (8.0–12.0 Hz) and theta (4.0–7.9 Hz) power spectra during PVT were calculated using fast Fourier transformation (FFT; frequency resolution, 0.97 Hz) with a Hamming window. FFT was performed using the data of each stimulus epoch (i.e., 5–10, 15–20, 25–30, and 35–40 min epochs from the start). The data of the first 30 seconds in each epoch were omitted to avoid startle responses. In total, the data of 270 sec in the stimuli epochs were used for FFT. The analysis was conducted with commercial software (CSA Sleep Analysis, version 1.16, NoruPro Light Systems, Inc., Japan). FFT was applied to overlapping (by 0.024 sec) EEG segments of 1.024 sec and was subsequently averaged for one 270 sec epoch. Artifacts in the EEG were removed using high-pass (0.5 Hz) and low-pass (30 Hz) digital filters.

### Rated sleepiness

The 9-point Karolinska Sleepiness Scale (KSS) [10, 15] was used. The participants rated their degree of sleepiness on a scale that included 1 (*very alert*), 3 (*alert*), 5 (*neither alert nor sleepy*), 7 (*sleepy*, *but not fighting sleep*), and 9 (*very sleepy*, *fighting sleep*) circling the number that represented their sleepiness. The KSS was administered at the end of the PVT. Participants recalled their subjective sleepiness *during* the task and scored it using KSS.

### Rated self-relevance to the stimuli

Participants were asked to evaluate the stimuli presented in the stimuli epochs using a 9-point scale that included 1 (*very relevant*), 3 (*relevant*), 5 (*neither relevant nor irrelevant*), 7 (*irrelevant*), and 9 (*utterly irrelevant*). Rating of self-relevance of the stimuli was performed at the end of each PVT task (i.e., every 40 min).

### Statistical analysis

A repeated-measures analysis of variance (ANOVA) was conducted with data on “Condition (Name, Other-name, and Cont conditions)” × “Time (first 20 and second 20 min blocks).” Degrees of freedom greater than 1 were reduced by the Huynh-Feldt *ε* correction to control for Type 1 error associated with violation of the sphericity assumption. Post-hoc analysis was performed by paired *t*-tests. Subjective sleepiness and self-relevance was compared by paired *t*-test. All statistical analyses were performed using the SPSS system for Mac, version 25.0.

## Results

The results of all the ANOVA’s are summarized in Table 1 In addition, the results of paired t-tests are described below.

**Table 1:** Results of ANOVA.

		F	DF	*p*	η^2^	Huynh-Feldt ε
RT	Cond	1.28	2, 58	n.s.	0.04	1.00
	Time	17.74	1, 29	< 0.01	0.38	1.00
	Cond * Time	1.89	2, 58	n.s.	0.06	1.00
CV	Cond	0.74	2, 58	n.s.	0.02	0.82
	Time	15.68	1, 29	< 0.01	0.35	1.00
	Cond * Time	0.96	2, 58	n.s.	0.03	0.99
Lapse (> 2.5 SD)	Cond	0.16	2, 58	n.s.	0.01	0.86
	Time	9.23	1, 29	< 0.01	0.24	1.00
	Cond * Time	0.49	2, 58	n.s.	0.01	0.83
Alpha power	Cond	3.06	2, 58	< 0.05	0.09	1.00
	Time	42.87	1, 29	< 0.01	0.59	1.00
	Cond * Time	2.43	2, 58	< 0.10	0.07	0.87
Theta power	Cond	0.2	2, 58	n.s.	< 0.01	1.00
	Time	1.52	1, 29	n.s.	0.05	1.00
	Cond * Time	0.95	2, 58	n.s.	0.03	1.00
KSS	Cond	0.37	2, 58	n.s.	0.01	0.88
Self-relevance	Cond	71.7	2, 58	< 0.01	0.71	0.95

RT: reaction time, CV: coefficient of variation, SD: standard deviation, KSS: Karolinska sleepiness scale.

### Subjective ratings

Self-relevance scores were higher in the Name (7.8, SD = 1.76) than Other-name condition (5.1, SD = 1.81) (*t* (29) = 5.11, *p* < 0.01). There was no significant difference in subjective sleepiness during the task among the conditions (5.9, SD = 2.47 for Name; 5.7, SD = 2.21 for Other-name; 6.1. SD = 2.47 for Cont conditions).

### PVT

**Fig 3:**
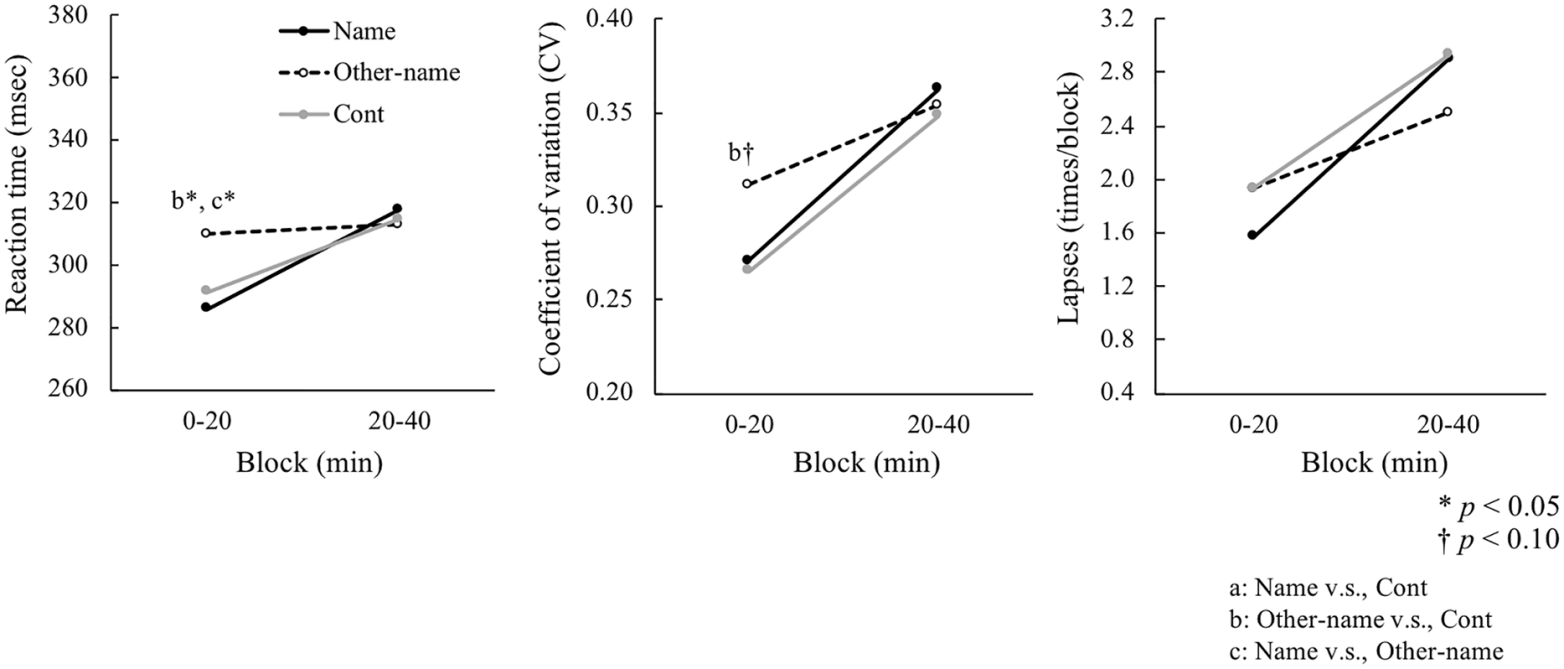
Reaction time (left), coefficients of variation (middle) and lapses (right) in the Psychomotor Vigilance Test. Name = name condition, Other-name = other’s name condition, and Cont = control condition (no sound), PVT: Psychomotor Vigilance Test, CV: coefficient of variation.

As to the reaction time (RT), there were no significant main effects of condition (*F*(2, 58) = 1.28, *p* = 0.28, η^2^ = 0.04) or interaction (*F*(2, 58) = 1.89, *p* = 0.15, η^2^ = 0.06). Although there were no significant effects observed in ANOVA, we performed the paired *t*-test for RT because there seemed differences between the conditions in the first block data. As shown in Fig 3-left, RT in the first 20 min block was longer in the Other-name condition compared to the Cont (*t* (29) = 2.24, *p* < 0.05) and Name (*t* (29) = 2.15, *p* < 0.05) conditions. There was no significant difference between the Name and Cont conditions in the first block (*t* (29) = 0.22, *p* = 0.82). Also, no significant effects were found in the second 20 min block between the conditions (*ps* > 0.63).

In CV, the main effects of condition (*F*(2, 58) = 0.74, *p* = 0.45, η^2^ = 0.02) and interaction (*F*(2, 58) = 0.96, *p* = 0.38, η^2^ = 0.03) were not significant. As shown in Fig 3-middle, however, CV was higher in the first 20 min block of the Other-name condition compared to the Cont condition (*t* (29) = 1.73, *p* < 0.10), suggesting that reaction times in the Other-name condition in the first 20 min block fluctuated more than in the Cont condition. There was no significant difference between the Name and Cont conditions in the first block (*t* (29) = 1.48, *p* = 0.14). Also, no significant effects were found in the second 20 min block between the conditions (*ps* > 0.30).

The number of lapses did not show any significant main effect (*F*(2, 58) = 0.16, *p* = 0.81, η^2^ = 0.01) and interaction (*F*(2, 58) = 0.49, *p* = 0.58, η^2^ = 0.01). The paired *t*-test showed no significant results between conditions in both the first and second blocks (*ps* > 0.26). All the data of PVT are shown in S1 Table.

### Spectral power density of EEG

**Fig 4:**
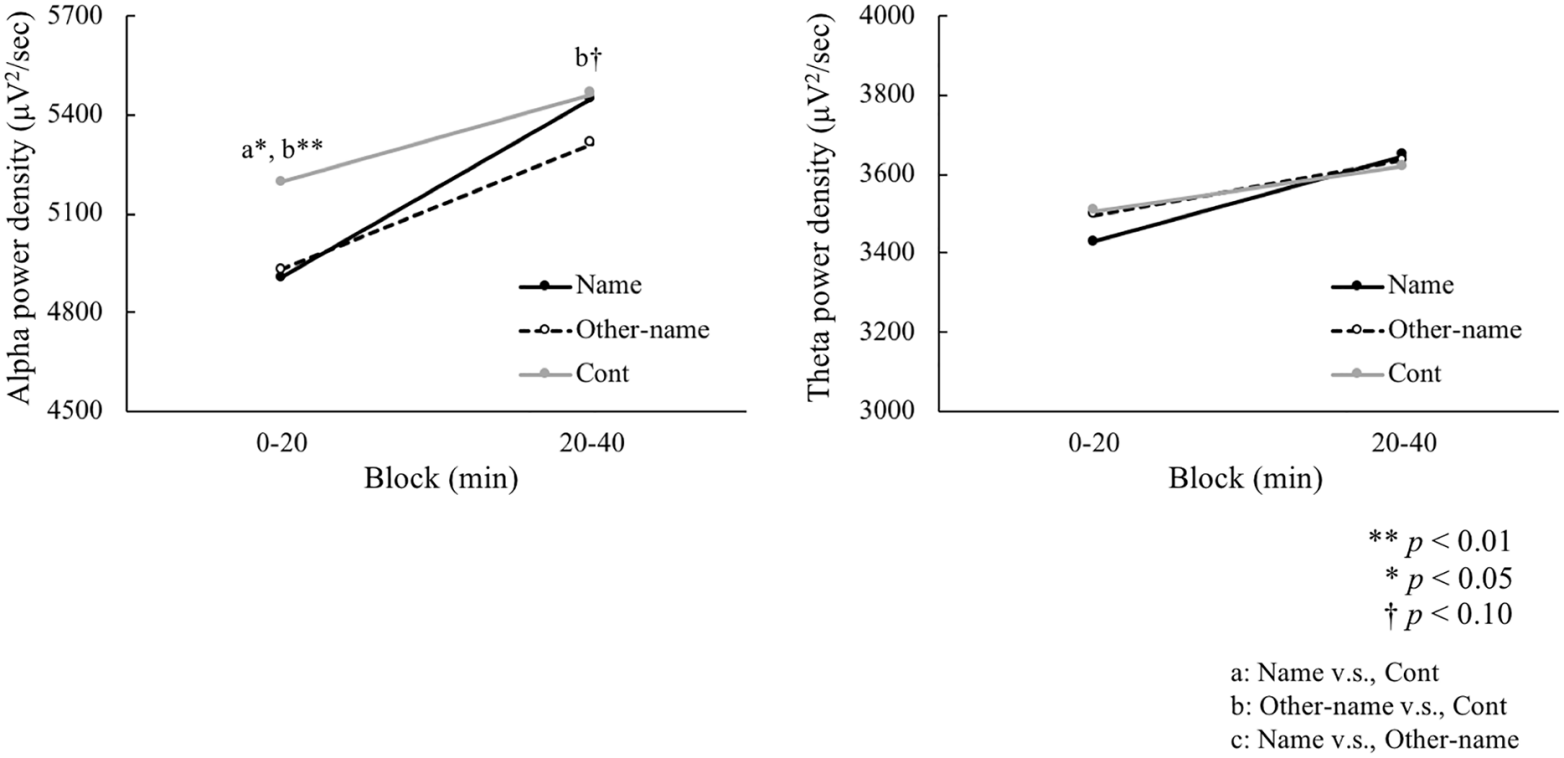
Spectral power densities in alpha (left) and theta (right) band frequencies. Name = name condition, Other-name = other’s name condition, and Cont = control condition (no sound).

The main effect of condition on EEG alpha spectral power density was significant (*F*(2, 58) = 0.06, *p* < 0.05, η^2^ = 0.09), as shown in Fig 4. Moreover, the interaction between condition and time was marginally significant (*F*(2, 58) = 2.43, *p* = 0.10, η^2^ = 0.07). The paired t-test indicated a significant reduction of alpha power density in the first 20 min blocks. The paired t-test showed that the arousal level was higher in the Name condition (*t* (29) = 2.40, *p* < 0.05) and the Other-name condition (*t* (29) = 2.64, *p* < 0.01) than in the Cont condition. The alpha power density in the second 20 min block was lower in the Other-name condition than in the Cont condition (*t* (29) = 1.70, *p* < 0.10). No significant effect was found between the Name and Cont conditions (*t* (29) = 0.17, *p* = 0.86) and between the Name and Other-name conditions (*t* (29) = 1.48, *p* = 0.15) in the second block.

As shown in Fig 4-right, an ANOVA indicated no significant main effect of condition (*F*(2, 58) = 0.20, *p* = 0.82, η^2^ = 0.01) and interaction (*F*(2, 58) = 0.95, *p* = 0.39, η^2^ = 0.03) in theta power densities.

## Discussion

The original finding of the present study is that the dissociation between performance and physiological arousal in the Other-name condition, that is, less self-relevant stimuli (i.e., other’s name) decreased performance compared to the Cont condition and simultaneously increased arousal, which was measured by EEG. In the Name condition, self-relevant stimulus (i.e., own name) resulted in shorter RT compared to the Other-name condition and increased arousal compared to the Cont condition, similar to a previous study [9]. These results suggest that experiencing less self-relevant stimuli deteriorated the performance of a monotonous task, which was not the case in self-relevant stimuli such as the own name.

The distractive effect on performance was observed in the Other-name condition, which was not the case in the Name condition. This is consistent with previous studies that have reported the facilitative effects of sounds on reaction times [7, 16], which are independent of the informational context of the sound regarding time or probability of target occurrence [17]. It is known that the sounds such as own name act as an unspecific alerting signal and induce an arousal effect as part of the orienting response generated towards attention-capture of auditory stimuli [18]. Attentional resources might therefore be concentrated on task performance in the Name condition, which might facilitate preparation for performing the task and actual task performance. In the Other-name condition, however, attentional resources might be used for identifying stimuli instead of preparing for the task. This could be a cause of the performance deterioration (i.e., prolonged RT, large fluctuation of CV) in the Other-name condition. It is also possible that performance improves when the facilitative effect of self-relevant sounds is stronger than the distractive effects.

In the present study, we did not control the presentation of auditory events (every 20 seconds) and the targets (inter-stimulus interval 1–10 seconds). That is, this study examined the effect of auditory events in the form of own or other’s name on performance in preparatory processes during the inter-stimulus interval, or preparatory interval. Our results suggest that own name let a person ready for response while other’s name impede the readiness. This may be because less self-relevant stimulus would compete with cognitive processing then prolong or fluctuate reaction time. The amount of attentional resources which is consumed for irrelevant stimuli could depend on the salience of the stimulation.

It is known that concurrent auditory stimulation during target processing is highly intrusive [4] and thus probably reduces attentional resources for target processing. However, the previous studies employed pure-tone to examine this matter, and it has not been studied from a self-relevance perspective or using auditory stimuli that have specific meanings or contexts like person’s names. This is an interesting topic for future research.

The facilitative and distractive effects of more and less self-relevant sounds could dissipate within 20 min because performance returned to the same level as in the Cont condition during the second 20-min epoch in both the Name and Other-name conditions. These results support previous reporting that the repeated presentation of distracting sounds decreased attentional orienting [19].

Given that alpha power density was reduced in both the Name and Other-name conditions, performance should improve equally in both conditions. However, the fluctuation of RT in the Other-name condition increased, whereas the same effect was not observed in the Name condition. The difference between these two conditions could be explained by attentional resources deployed for executing the current task. Task performance is expected to improve when more attentional resources are deployed to execute the current task. Moreover, performance is expected to improve through the arousal effect of stimuli, if the amount of attentional resources increases with the increase in the arousal level. This suggests that increased attentional resources increased by stimuli in the Other-name condition were deployed for something other than executing the task, which might have impeded performance regardless of the increases in attentional resources.

One possible explanation for these findings could be that less self-relevant stimuli induce mind-wandering. Mind-wandering is defined as the occurrence of self-generated thoughts that are both unrelated to the current task and decoupled from immediate sensory perceptions [20, 21]. It is known that mind-wandering caught by a probe distracts attention to stimuli in an ongoing task, which was associated with longer reaction times [20]. Thoughts unrelated to the task might have occurred in the Other-name condition which could have consumed attentional resources that would have been deployed to the ongoing task. This would be an interesting topic for future research.

There are certain limitations to the present study. First, the timing between the auditory events (at predictable 20 seconds intervals) and the PVT (at unpredictable 2–10 seconds intervals) targets were not synchronized. If the sound was presented immediately before the target stimuli, the statistical results might have been more precise [3, 22]. On the other hand, such control of the sound presentation would have decreased the ecological validity of the study because it would not reflect real-life settings such as driving, air-traffic control, or a night watch among others. We prioritize the ecological validity of this study so that the results would be more closely related to real-life work situations. Second, the number of participants in this study was small for detecting subtle difference between the conditions. Third, a technique for improving the sensitivity of detection, such as the performance speed cumulative distributive function (CDF) would have been useful [23], however, it requires expert calculation skills. Finally, we did not have a pure-tone condition because our previous study [9] had indicated that a pure-tone and other-names had the same effect (i.e., no significant effect on RT and EEG). Nevertheless, including a pure-tone condition might have provided clearer results by facilitating a strict comparison of different types of sound stimuli. These issues be considered in future studies.

In conclusion, based on the overall pattern of results, is that (1) arousal increased by hearing a name, regardless of its self-relevance, and (2) hearing less self-relevant stimuli such as other’s name had a distractive effect on ongoing task performance, although it increased arousal. The results of the behavioral effects, however, are relatively small and not consistently supported by all of the performance indicators, being aware that further experiments are urgently necessary.

## Supporting information

S1 Table Data of performance. (DOCX)
