# Supplementary material for: Hearing own or other’s name has different effects on monotonous task performance
Source: PLoS One. 2018 Sep 26;13(9):e0203966. doi: 10.1371/journal.pone.0203966 (PMC6157865; doi:10.1371/journal.pone.0203966)
Supplement: S1 Table — (DOCX) [file pone.0203966.s001.docx]

| S1 Table. Data of performance | | |  |  |  |  |  |  |
| --- | --- | --- | --- | --- | --- | --- | --- | --- |
|  |  | Condition |  | First block | SD | Second block | SD |  |
|  | RT | Name |  | 285.6 | 29.90 | 317.8 | 71.54 |  |
|  |  | Other-Name |  | 309.6 | 66.08 | 312.9 | 51.92 |  |
|  |  | Control |  | 291.3 | 48.59 | 314.5 | 64.90 |  |
|  | CV | Name |  | 0.27 | 0.111 | 0.36 | 0.235 |  |
|  |  | Other-Name |  | 0.31 | 0.184 | 0.35 | 0.172 |  |
|  |  | Control |  | 0.27 | 0.101 | 0.35 | 0.188 |  |
|  | Lapse (> 2.5 SD) | Name |  | 1.6 | 2.08 | 2.9 | 2.16 |  |
|  |  | Other-Name |  | 1.9 | 1.96 | 2.5 | 1.81 |  |
|  |  | Control |  | 1.9 | 2.16 | 2.9 | 3.19 |  |
| RT: reaction time, CV: coefficient of variation, SD: standard deviation. | | | | |  |  |  |  |
